# Epidemiology of respiratory syncytial virus in a large pediatric hospital in Central Italy and development of a forecasting model to predict the seasonal peak

**DOI:** 10.1186/s13052-024-01624-x

**Published:** 2024-04-08

**Authors:** Renato Cutrera, Marta Luisa Ciofi degli Atti, Andrea Dotta, Carmen D’Amore, Lucilla Ravà, Carlo Federico Perno, Alberto Villani

**Affiliations:** 1 Pediatric Pulmonology and Cystic Fibrosis Unit, Bambino Gesù Children’s Hospital, IRCCS, Rome, Italy; 2 Epidemiology, Clinical Pathways and Clinical Risk Unit, Medical Direction, Bambino Gesù Children’s Hospital, IRCCS, Rome, Italy; 3 Neonatal Intensive Care Unit, Bambino Gesù Children’s Hospital, IRCCS, Rome, Italy; 4 Department of Diagnostic and Laboratory Medicine, Unit of Microbiology and Diagnostic Immunology, Multimodal Laboratory Medicine Research Area, Bambino Gesù Children’s Hospital, IRCCS, Rome, Italy; 5 Hospital University Pediatric Clinical Area, Bambino Gesù Children’s Hospital, IRCCS, Rome, Italy; 6 Systems Department, University of Rome Tor Vergata, Rome, Italy

**Keywords:** Respiratory Syncytial Virus, Children, SARIMA, Epidemiology, Hospitalization

## Abstract

**Background:**

Respiratory Syncytial Virus (RSV) is responsible for the majority of acute lower respiratory infections in infants and can affect also older age groups. Restrictions linked to the emergence of the SARS-CoV-2 pandemic and their subsequent lifting caused a change in the dynamics of RSV circulation. It is therefore fundamental to monitor RSV seasonal trends and to be able to predict its seasonal peak to be prepared to the next RSV epidemics.

**Methods:**

We performed a retrospective descriptive study on laboratory-confirmed RSV infections from Bambino Gesù Children’s Hospital in Rome from 1st January 2018 to 31st December 2022. Data on RSV-positive respiratory samples (*n* = 3,536) and RSV-confirmed hospitalizations (*n* = 1,895) on patients aged 0–18 years were analyzed. In addition to this, a SARIMA (Seasonal AutoRegressive Integrated Moving Average) forecasting model was developed to predict the next peak of RSV.

**Results:**

Findings show that, after the 2020 SARS-CoV-2 pandemic season, where RSV circulation was almost absent, RSV infections presented with an increased and anticipated peak compared to pre-pandemic seasons. While mostly targeting infants below 1 year of age, there was a proportional increase in RSV infections and hospitalizations in older age groups in the post-pandemic period. A forecasting model built using RSV weekly data from 2018 to 2022 predicted the RSV peaks of 2023, showing a reasonable level of accuracy (MAPE 33%). Additional analysis indicated that the peak of RSV cases is expected to be reached after 4–5 weeks from case doubling.

**Conclusion:**

Our study provides epidemiological evidence on the dynamics of RSV circulation before and after the COVID-19 pandemic. Our findings highlight the potential of combining surveillance and forecasting to promote preparedness for the next RSV epidemics.

**Supplementary Information:**

The online version contains supplementary material available at 10.1186/s13052-024-01624-x.

## Background

Respiratory Syncytial Virus (RSV), a negative single-stranded RNA virus belonging to the family of Paramyxoviruses, genus Pneumoviridae [1, 2], is the most common cause of acute lower respiratory infections - mainly bronchiolitis - in the young pediatric population [3, 4]. Globally, it is responsible for almost 33.1 million cases per year in the population under 5 years old, with roughly 10% (3.2 million) of them resulting in hospitalizations [5]. Hospitalization rate is higher in children below 6 months of age, especially if born pre-term [6]. Other risk factors for hospitalization include underlying chronic respiratory condition or congenital cardiopathy, immunodeficiencies or neuromuscular pathologies [6, 7]. RSV not only interests the pediatric population, but is a relevant cause of morbidity and mortality for patients above 65 and for immunocompromised patients [8].

While the diagnosis of bronchiolitis is clinical, nasopharyngeal swabs allow for the virus identification via molecular test (Polymerase Chain Reaction, PCR) or, with a lower accuracy, via rapid antigenic test. In the US, Centers for Disease Control and Prevention (CDC) monitor the national yearly circulation of RSV using diagnostic testing, and weekly report the frequency of positive results to the National Respiratory and Enteric Virus Surveillance System (NREVSS), making data on RSV circulation trends available [9]. In Italy only a few hospitals have a virus surveillance system [10, 11] and a routine national monitoring system is lacking.

In 2020, SARS-CoV-2 pandemic determined relevant changes in the trend of RSV infection curve. Restrictive measures introduced to limit SARS-CoV-2 spreading, such as social distancing, abolition of gatherings, mandatory face mask wearing, together with frequent hand washing, disinfectant use and fear of contagion led to a drastic reduction of main pediatric illnesses due to common seasonal viruses, including RSV [12–15]. During the pandemic (late 2020) a drastic reduction of RSV-caused bronchiolitis was observed [16–18]. COVID-19 pandemic was also associated with an increased age of RSV infection [19, 20]. With the progressive elimination of SARS-CoV-2 related restrictions, there was a resurgence of seasonal epidemics associated with respiratory viral infections in children [21]. Several European countries reported out-of-season RSV outbreaks during the spring of 2021 [21–23], followed by RSV epidemic in autumn of the same year. RSV infections cause a higher proportion of hospitalization and a greater use of respiratory support (HNFC and CPAP) compared to other viruses during autumn–winter 2022–2023 [21]. Considering the high incidence of RSV infection, it is fundamental to gather data on the RSV dynamics to prevent a future RSV epidemic.

The present study aims to describe the epidemiological trend of infections and hospitalizations of laboratory confirmed RSV-bronchiolitis in the past 5 years at Bambino Gesù Children’s Hospital (Rome) and to develop a forecasting model based on data from previous seasons to predict the peak week of RSV hospitalizations for the next season.

## Methods

### Setting

This study was conducted at Bambino Gesù Children’s Hospital (Ospedale Pediatrico Bambino Gesù, hereafter OPBG), a 607-bed tertiary care academic hospital in the Lazio Region, Italy. The emergency department (ED) provides free urgent medical care to the pediatric population on a 24/7 basis; ED admissions were 85,012 in 2018, 89,558 in 2019, 62,010 in 2020, 79,624 in 2021 and 100,030 in 2022. Hospital admissions were 28,754 in 2018, 29,432 in 2019, 26,178 in 2020, 27,963 in 2021 and 28,980 in 2022.

### Study design and population

This was a retrospective descriptive study including all patients aged between 0 and 18 years with RSV infections confirmed with molecular or antigenic tests from 1st January 2018 to 31st December 2022.

To describe the trend in RSV-related hospitalizations over this study period, only patients urgently hospitalized with a laboratory-confirmed RSV infection within 48 h from emergency visit date were analyzed.

### Virological data

RSV was detected with molecular testing (RT-PCR multiplex, BioFire Filmarray Respiratory Panel 2.1 and Allplex Respiratory Panel Assays) and/or antigenic testing (Binax now RSV and SD-Biosensor).

### Clinical data

The demographic and clinical information of children who accessed the ED and were hospitalized from 1st January 2018 to 31st December 2022 was retrospectively extracted from the electronic health records of OPBG. In detail, information on ED visits was collected from the OPBG Healthcare Emergency Information System (HEIS), which includes patient demographics, ICD-9-CM diagnosis and status at discharge (i.e. hospitalized, or discharged at home). Acute Respiratory Infections (ARI) were defined according to the ICD-9 CM diagnosis at discharge (see Supplementary File 1). Criteria for admission to ICUs included respiratory failure requiring mechanical ventilation, or risk of severe acute deterioration or hemodynamic instability.

Information on RSV results of respiratory samples (i.e., naso-pharyngeal swabs, tracheal swabs and/or broncho-alveolar lavages) obtained in the same time period from OPBG patients was derived from the Hospital Electronic Laboratory Information System. Respiratory samples testing RSV-positive within 3 months were excluded from the analysis.

RSV testing was ordered by attending clinicians, as of hospital guidelines developed by multidisciplinary teams and implemented since 2014 [24, 25].

### Statistical analysis and modelling

The number of RSV-confirmed infections, the number of RSV-related hospitalizations and intensive care unit (ICU) admissions were summarized either by year or epidemic seasons (each season starting from week 39 of the previous year and ending at week 12 of the next year) and by week, and were expressed as counts and proportions. Seasons 2017-18 and 2022-23 were incomplete, lacking the final weeks of 2017 and the initial weeks of 2023, respectively, while the other 4 seasons were representative of the whole season (2018-19, 2019-20, 2020-21, 2021-22).

Descriptive analysis was conducted, stratifying for age range (< 1 year, 1–4 years, 5–9 years and > 10 years) and for severity (ICU admissions). All data were anonymized and presented exclusively as aggregates. Significance in trend was tested using Cochrane Armitage test.

Seasonal Autoregressive Integrated Moving Average, SARIMA or Seasonal ARIMA, is a forecasting method that supports univariate time series data with a seasonal component. It includes three parameters to specify the autoregression (AR), differencing (I) and moving average (MA) for the seasonal component of the series, as well as an additional parameter for the period of the seasonality (S).

SARIMA forecasting model was used to predict the week of the peak of RSV hospitalizations in 2023, based on the weekly number of RSV hospitalizations of the previous years (2018–2022). The general form of seasonal model is SARIMA (p, d, q) (P, D, Q) where parameter 𝑝 and seasonal P represent the periods to lag for, d and seasonal D represent the number of differencing transformations done to remove trend and/or seasonality therefore turning the time-series into a stationary one, q and seasonal Q represents the lag of the error component of the ARIMA model. Mean Absolute Percentage Error (MAPE) was used to measure forecast accuracy [26], since it measures how far off the prediction is from the actual observed cases.

The forecasting model yields plausible prediction values when MAPE is low. Lewis [27] summarized the criteria of MAPE as follows: a MAPE value of less than 10% indicates excellent predictive accuracy, a value between 10 and 20% indicates good predictive ability, a value between 20 and 50% is acceptable accuracy, and a value over 50% indicates unacceptable predictive accuracy.

Proportion of ARI ED admissions that were tested for RSV was included in SARIMA model as exogenous variable, in order to consider for RSV testing propensity. A sensitivity analysis was performed for comparing the ED admission forecasts resulting from SARIMA models with and without exogenous variable. Likelihood-ratio test was conducted to test differences between the two models.

All statistical analyses were performed using STATA, Statistical Software: Release 17 (StataCorp LP, College Station, TX).

### Ethical considerations

The study was approved by OPBG Ethical Committee. Consent was waived due to the retrospective nature of the investigation.

## Results

### Weekly trend of RSV infections

During the study period, a total of 3,536 confirmed cases of RSV infections were considered. Out of 3,536 confirmed cases of respiratory syncytial virus infection, 3,397 (96.1%) were diagnosed through molecular method and 139 (3.9%) through antigen test. Out of 3,397 cases diagnosed with molecular test, 32,0% (*n* = 1,088) were performed in Emergency Department.

**Fig. 1:**
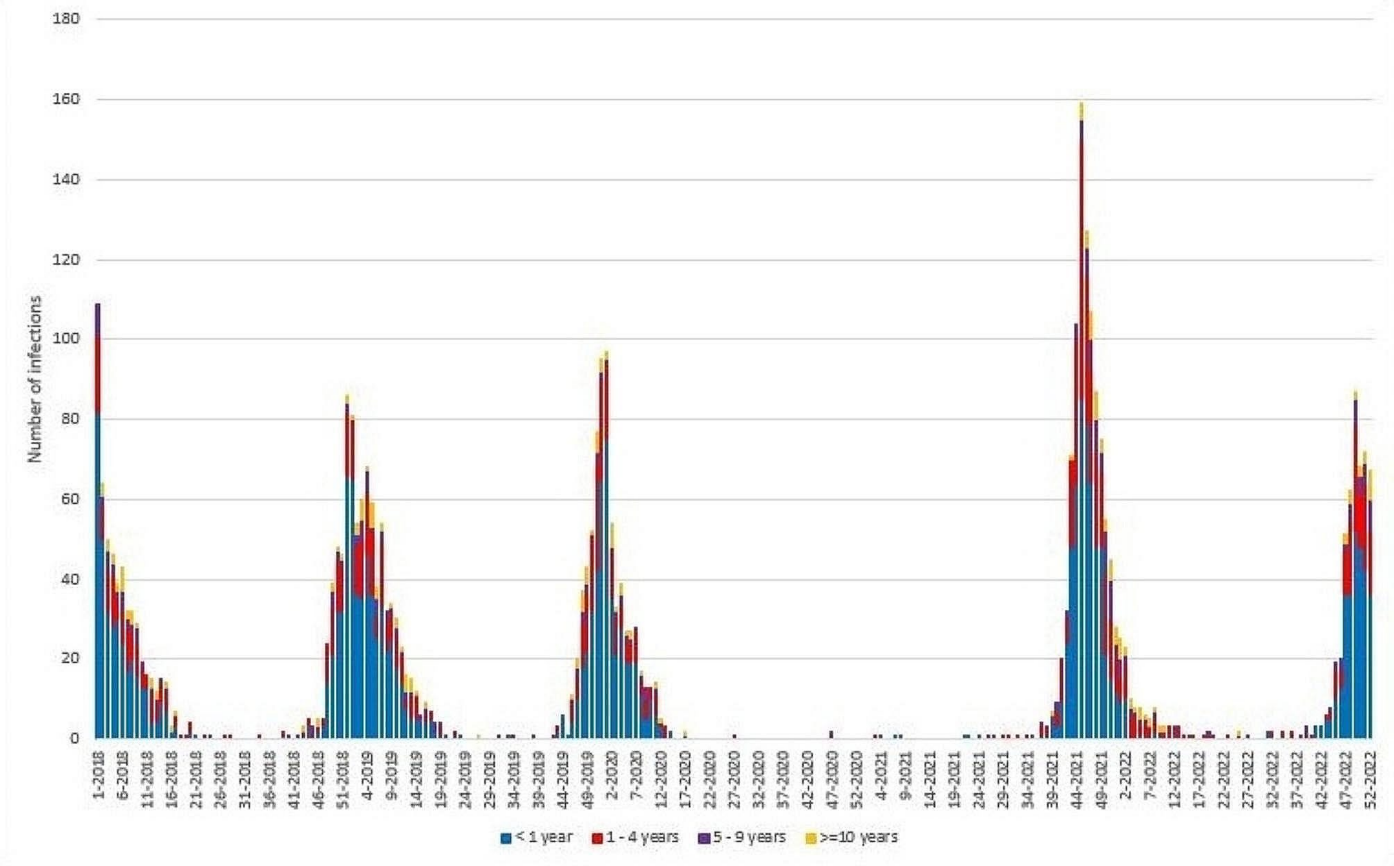
Weekly trend of laboratory-confirmed RSV infections; OPBG, 2018–2022

Figure 1 shows the weekly trend of RSV infections during the study period (2018–2022), stratified for age classes. Peaks of RSV infections showed a regular pattern during the pre-pandemic period. The beginning of the SARS-CoV-2 pandemic in autumn 2020 marked a striking decrease of RSV infections during the 2020–2021 season. In October-November 2021 (weeks 43/44-2021), a new, anticipated RSV season started, showing an early and more elevated peak compared to previous seasons. During 2022, a peak similar to the one of 2018 was observed.

Table 1 summarizes data on RSV infections stratified by year and age classes. RSV infections were inversely proportional to age, with the majority of infections diagnosed in infants < 1 year of age (*n* = 2,187; 61.8%) followed by children aged 1–4 years (*n* = 998; 28.2%); RSV infections diagnosed in children aged ≥ 5 years accounted for about 10% of cases (*n* = 351). A statistically significant proportional increase of cases in children of older age groups (1–4 and 5–9 years; *p* ≤ 0.01) was observed ranging from 24.2% to 5.4% in 2018 to 30.0% and 8.7% in 2022, respectively; on the contrary a significant decrease of RSV infections in patients < 1 year old from 66.3% (*n* = 338) in 2018 to 56.2% (*n* = 318; *p* = 0.001) in 2022 was observed. Findings were similar when looking at RSV epidemic seasons (Supplementary File 2): again, season 2020-21 showed a lack of RSV circulation, in concomitance with the pandemic period; season 2021-22 showed instead an increased peak of infections. For all included seasons, most infections were reported for infants in their first year of age, and a proportional increase of infections in children of older age groups was again observed in the post-pandemic seasons.

**Table 1:** Trend of laboratory-confirmed RSV infections by year and age-classes; OPBG, January 2018– December 2022. Infections were confirmed via either antigenic or PCR tests

	2018 N (%)	2019 N (%)	2020 N (%)	2021 N (%)	2022 N (%)	Total N (%)	*P*-value*
**Age classes**							
< 1	538 (66.3%)	592 (63.9)	226 (67.5)	513 (57.2)	318 (56.2)	2,187 (61.8)	< 0.001
1–4	196 (24.2)	249 (26.9)	84 (25.1)	299 (33.3)	170 (30.0)	998 (28.2)	0.0002
5–9	44 (5.4)	46 (5.0)	15 (4.5)	54 (6.0)	49 (8.7)	208 (5.9)	0.01
≥ 10	33 (4.1)	40 (4.3)	10 (2.9)	31 (3.5)	29 (5.1)	143 (4.0)	0.8
Total	**811** **(100.0)**	**927** **(100.0)**	**335** **(100.0)**	**897** **(100.0)**	**566** **(100.0)**	**3,536** **(100.0)**	

* *Cochrane Armitage test for trend*

### Weekly trend of RSV hospitalizations and ICU admissions

**Fig. 2:**
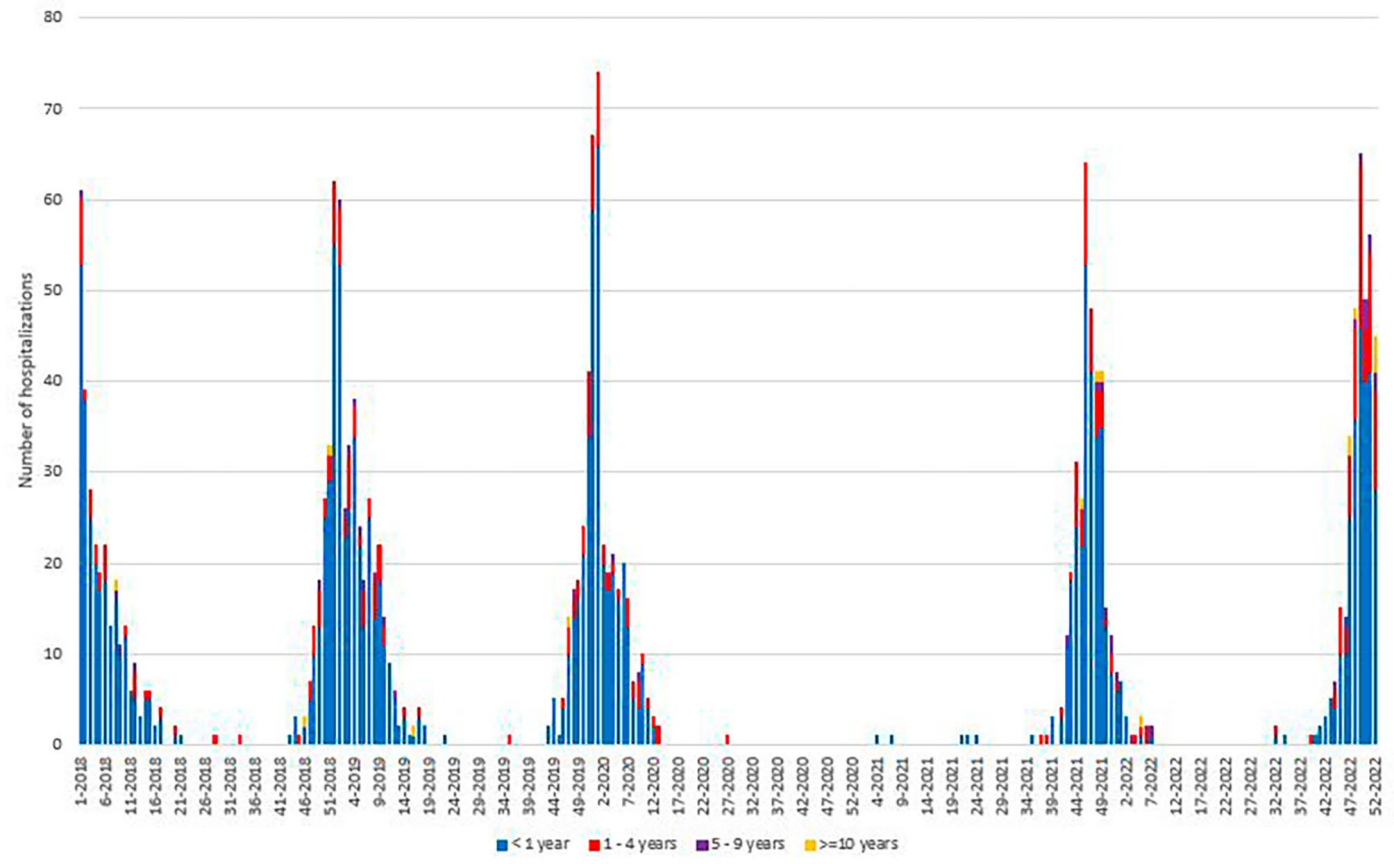
Weekly trend of RSV-related hospitalizations; OPBG, 2018–2022

As depicted in Fig. 2, the weekly trend of hospitalizations is consistent with the weekly trend of infections. The total number of hospitalizations during the study period was 1,895 and 82,7% of those occurred in infants below 1 year of age (Table 2). Similarly to what was found for RSV infections, trend of weekly RSV hospitalizations showed a lack of hospitalizations during the SARS-CoV-2 pandemic, followed by an anticipated post-pandemic peak (week 46-2021) compared to the usual pre-pandemic peaks (occurring in weeks 52-2018 to 1-week 2019 and in weeks 52-2019 to 1-2020) (Fig. 2). However, during 2022 a proportional decrease of hospitalizations of children below 1 year was observed, and that was matched by a proportional increase of hospitalizations in the 1–4 years group. A smaller but significant proportional increase of hospitalizations was also observed in the 5–9 years group (Table 2).

**Table 2:** Trend of RSV-related hospitalizations by year and age-classes; OPBG, January 2018– December 2022

	2018 N (%)	2019 N (%)	2020 N (%)	2021 N (%)	2022 N (%)	Total N (%)	*P*-value*
**Age classes**							
< 1	408 (86.8)	449 (85.4)	165 (85.5)	280 (82.6)	265 (72.2)	1,567 (82.7)	< 0.001
1–4	53 (11.3)	65 (12.4)	26 (13.5)	49 (14.4)	81 (22.1)	274 (14.5)	< 0.001
5–9	6 (1.3)	10 (1.9)	2 (1.0)	7 (2.1)	13 (3.5)	38 (2.0)	0.04
≥ 10	3 (0.6)	2 (0.4)	0 (0.0)	3 (0.9)	8 (2.2)	16 (0.8)	0.01
**Total**	**470** **(100.0)**	**526** **(100.0)**	**193** **(100.0)**	**339** **(100.0)**	**367** **(100.0)**	**1,895** **(100.0)**	

*Cochrane Armitage test for trend

Similarly, seasonal data (Supplementary File 3) showed an almost complete lack of hospitalizations during season 2020-21, while, in the two previous seasons, hospitalizations were, respectively, 462 (2018-19) and 417 (2019-20), ≥ 85% of them in infants below one in both seasons. During the post-pandemic period, we observed 350 hospitalizations (season 2021-22), about 82% of them in children below one year of age. An age-dependent statistically significant variation in hospitalizations, with a proportional decrease in the smaller age group and a proportional increase in children aged 1–4 years old and ≥ 10 years old for the seasons after the pandemic was identified. (Supplementary File 3).

Severity of RSV cases was assessed looking at ICU admissions: the percentage of ICU admissions among patients hospitalized for RSV was 12.4% as, in total, 235 ICU hospitalizations were registered during the study period (Table 3). (Supplementary File 4).

**Table 3:** Trend of RSV-related ICU admissions by year and age-classes; OPBG, January 2018– December 2022

	2018 N (%)	2019 N (%)	2020 N (%)	2021 N (%)	2022 N (%)	Total N (%)	*P*-value*
**Age classes**							
< 1	53 (94.6)	55 (94.8)	21 (95.5)	34 (89.5)	51 (83.6)	**214** **(91.1)**	0.02
1–4	3 (5.4)	3 (5.2)	1 (4.5)	3 (7.9)	9 (14.7)	**19** **(8.1)**	0.05
5–9	0 (0.0)	0 (0.0)	0 (0.0)	1 (2.6)	1 (1.6)	**2** **(0.8)**	0.2
≥ 10	0 (0.0)	0 (0.0)	0 (0.0)	0 (0.0)	0 (0.0)	**0** **(0.0)**	-
**Total**	**56** (100.0)	**58** (100.0)	**22** (100.0)	**38** (100.0)	**61** (100.0)	**235** (100.0)	

* *Cochrane Armitage test for trend*

### Development of a forecasting model to predict the peak of RSV hospitalizations

**Fig. 3:**
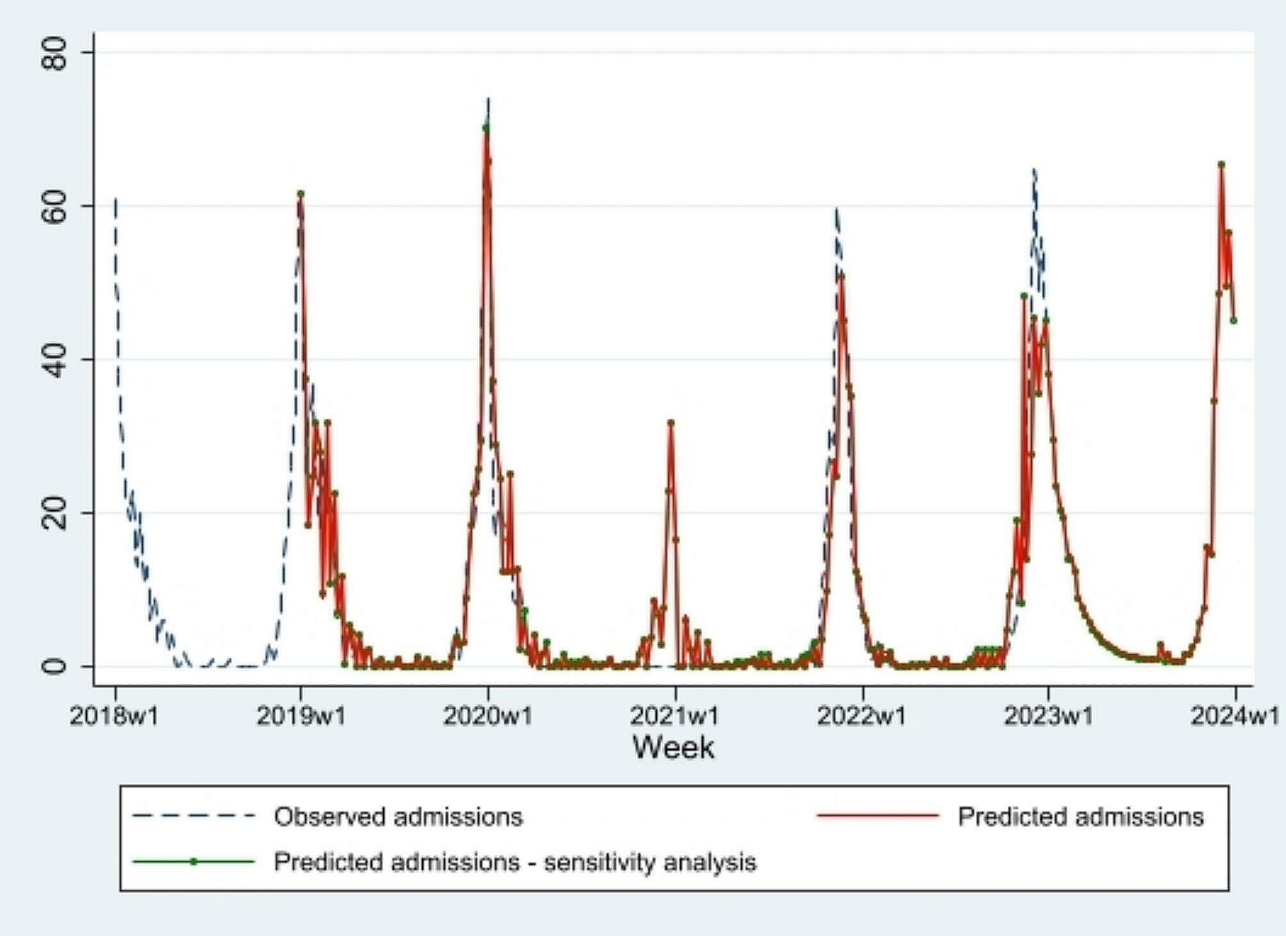
Predictions of RSV-related hospitalizations in 2023, OPBG

A model for predicting the week of the peak of RSV hospitalizations was built; weekly data from 2018 to 2022 were used to predict the peak of RSV-related hospitalizations in season 2023–2024, which was forecasted to occur in week 49 in 2023. Predicted RSV-related hospitalizations were in line with those observed before the SARS-CoV-2 pandemic period. During the pandemic period a peak of hospitalizations was expected and predicted by the SARIMA model; in the post-pandemic period a slight difference was observed between predicted and observed hospitalizations; peaks of observed hospitalizations were either anticipated or delayed, in seasons 2021–2022 and 2022-23, respectively, with respect to predicted peaks (Fig. 3). The accuracy of the prediction model was reasonable with a MAPE of 33%.

Proportion of ARI ED admissions that were tested for RSV did not show statistically significant variations over the study years, being 10.0% (1,711/17,029) in 2018, 9.5% (1,775/18,717) in 2019, 8.5% (1,070/12,551) in 2020, 10.7% (1,723/16,070) in 2021 and 11.3% (2,051/18,186) in 2022 (*p* = 0.4). Sensitivity analysis showed that propensity to RSV testing did not affect RSV-related hospitalizations forecast (Fig. 3). The accuracy of the prediction model including RSV testing propensity remained unchanged. No statistically significant differences were observed between the two models (*p* = 0.4).

**Fig. 4:**
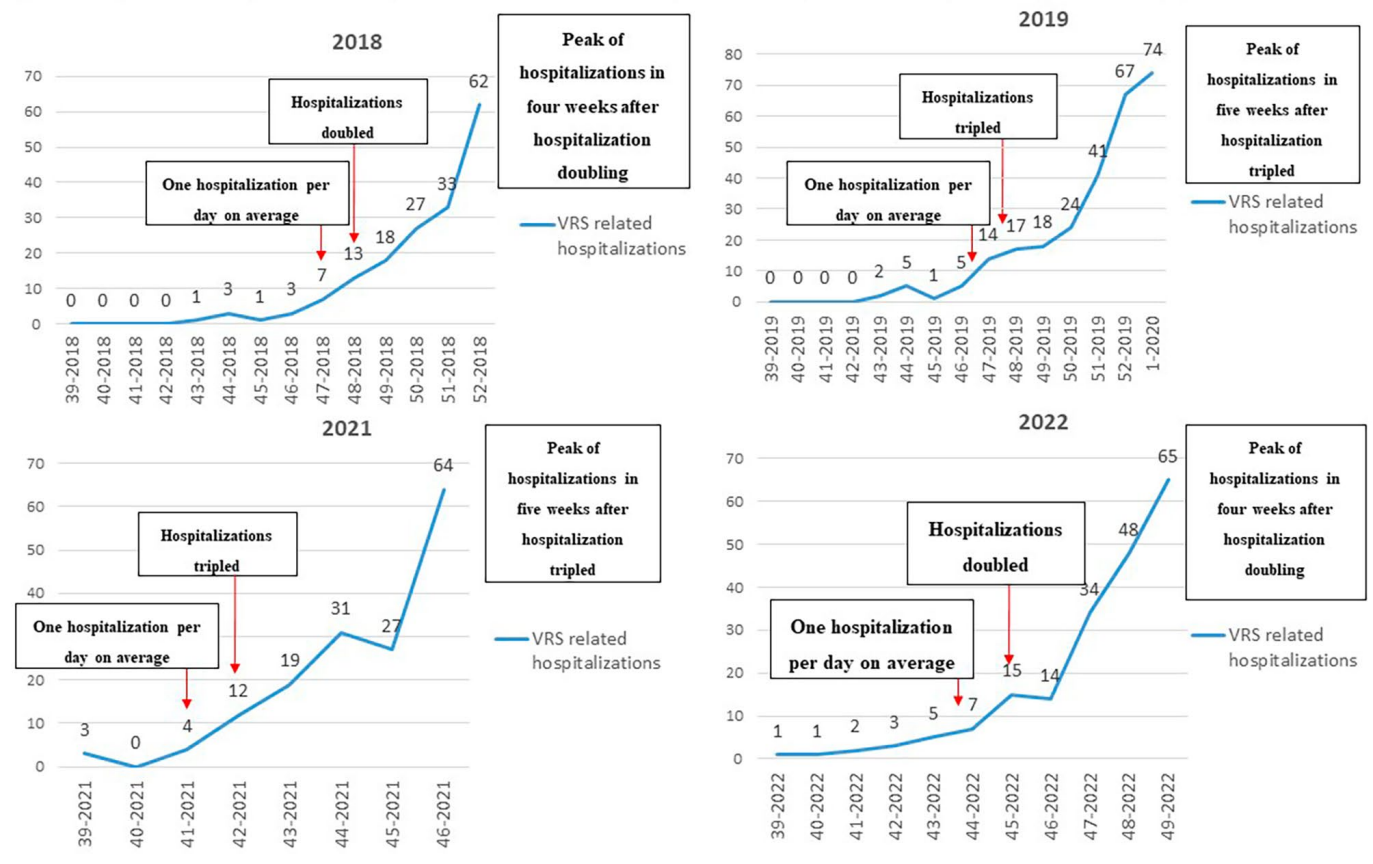
Analysis of the weekly increase of hospitalizations from the start of the epidemic season (39-week) to the peak of hospitalizations, by year

In addition to this, an analysis of the weekly increase of hospitalizations from the start of the epidemic season has been conducted. Starting from the week when, on average, one RSV-related hospitalization per day occurs, the next week usually showed a doubling or tripling of hospitalizations: this represented a landmark for the prediction of the peak of RSV hospitalizations, as it always occurred within 4–5 weeks from this hospitalization doubling/tripling (Fig. 4).

## Discussion

This study presented the epidemiological trend of infections and hospitalizations of laboratory confirmed RSV-cases in pediatric patients from 0 to 18 years old during 2018–2022 in a single tertiary care academic hospital pediatric hospital in Italy, with a very high access to emergency department and to ward admission.

Findings showed that, while the curve of infections and hospitalizations was pretty constant during the pre-SARS-CoV-2 period, the insurgence of the pandemic in the autumn-winter 2020 led to a drastic reduction of RSV infections and RSV related hospitalizations. In the post-pandemic period, an increased and anticipated peak of RSV cases and hospitalizations was recorded. While the majority of RSV cases and hospitalizations was diagnosed in the youngest age group (under 1 year of age), the post-pandemic period revealed a statistically significant trend towards a proportional increase in cases and hospitalizations in older age groups, especially in the 1–4 years group. This might potentially be due to an increased susceptibility to RSV in previously unexposed young children during SARS-CoV-2 pandemic. Indeed, during the pre-COVID-19 era, 60–70% of children under one year of age, and almost all patients below two years had their first RSV infection, developing the required immunity for protection from severe infections [2, 3, 28, 29]. The prolonged period of low exposition to the pathogen of children and new mothers, with the lack of transmission of protective antibodies to the newborns, exposed children and newborns to a higher risk of contracting RSV and developing severe forms of the related disease [30]. These findings are in line with international evidence that the COVID-19 pandemic correlated with an increased age of RSV infection. In Lyon, France, mean age of RSV positive hospitalized children was 4.8 months in 2020/2021, compared to 2.2–3.1 months for the 2016/2017–2019/2020 period [19]. The same phenomenon was registered in Australia, where mean age of RSV positive children that required hospitalization or access to the emergency department was significantly higher in 2020 (18.4 months) compared to 2012–2019 time period (7.3–12.5 months) [20]. We found a small number of patients aged ≥ 10 years old requiring hospitalization, as expected given that RSV hospitalization risk decreases with age [31].

Our data on the dynamics of the RSV seasonality also concur with reported evidence worldwide. Usually, in the northern hemisphere, RSV season begins in November-December and ends in March, peaking during January-February– and this in line with our pre-pandemic data–, while it lasts generally from June to September in the southern hemisphere [32–34]. The SARS-CoV-2 pandemic in the northern hemisphere began coincidentally with the usual RSV epidemic peak, and adoption and implementation of restrictive measures determined the sudden early end of RSV epidemic season [12–17, 19]. In the US, after relaxation of restrictions, an off-season surge of cases was reported [35, 36], and it was forecasted that timing and intensity of reemergent RSV epidemics might be different from the usual, pre-pandemic RSV seasons [37]. We also observed an anticipated, increased peak of infections in 2021 compared with pre-pandemic data. In the southern hemisphere, a lack of cases of RSV infection was observed during winter (May-August 2020), while the virus started circulating again at the beginning of spring (mid-late August 2020), when usually the RSV season ends [20, 38]. The RSV-bronchiolitis peak, instead, was registered in 2021, substantially late compared with previous years, and with more severe cases, potentially because of the reduced exposure to the virus in the previous seasons [38, 39].

In light of the different dynamics of viral infections that arose with the COVID-19 pandemic, it is important to strengthen the implementation of non-pharmaceutical measures to reduce respiratory infections. In addition to non-pharmaceutical measures, the RSV prevention system is based on the administration of monoclonal antibodies (Palivizumab, Nirsevimab) in high-risk subjects [40, 41]. Monoclonal antibodies might, for example, being administered in older children, and it should be taken into account that the epidemic season might be temporally unusual. Recently, RSV vaccine safety and efficacy has been documented to protect infants up to 6 months of age [42–43].

In order to minimize the global impact of RSV and associated bronchiolitis, healthcare systems must be prepared in advance to the yearly virus circulation. For this reason, we developed a forecasting model to estimate the trend of RSV hospitalizations based on a time series forecasting model (SARIMA), which could also be useful at regional or national level. In fact, accurate prediction of the peak of RSV-bronchiolitis might call for the increase of ICU beds, for a greater availability of emergency visits on the territory, and for the correct management of the preventive measures.

Given the differences between pre- and post-pandemic RSV dynamics, it was important to define whether future RSV trends could be adequately predicted using data from previous years. Our model, developed using data from 2018 to 2022, showed a reasonable level of accuracy (MAPE = 33%, meaning that the model’s predictions are, on average, off by 33% from the real values). Even if RSV seasonality pattern may tend to realign to the pre-pandemic period, our modelling data suggest that the 2023–2024 season still has atypical seasonality, starting anticipatedly and ending earlier. Analyzing hospital data admission and virological results, we also showed that, after case doubling/tripling, the RSV peak can be expected within 4–5 weeks. These data should be easily available and can help hospitals predicting the increase of RSV-related hospitalizations.

Our findings might have some limitations. First, the retrospective observational nature of the study prevents from making causal inferences. While we considered ICU hospitalizations to assess severity, other indexes might be considered. A potential source of bias is that RSV infection was confirmed with different diagnostic modalities (either antigenic or PCR tests) and this might act as a confounding factor. In addition to this, we did not differentiate between RSV subtypes A and B, and we therefore could not identify eventual differences in subtypes circulation over the study period and in comparison with previous evidence [44]. Prediction of RSV-related hospitalizations with SARIMA model was based only on hospitalization data in previous years and did not consider other variables that could affect RSV epidemiology, such as meteorological conditions and RSV circulation in other countries. Including these variables in the prediction model could improve its prediction accuracy. Further modelling studies including exogenous variables should be conducted, and could evaluate the impact of RSV prevention measures, such as monoclonal antibodies [40, 41] and RSV vaccine to protect infants up to 6 months of age [42–43]. It is also important to highlight that we included pediatric patients (0–18 years), with confirmed RSV infection; included patients might have had coinfections, with RSV not being the only pathogen present, but we could not establish whether there was an association with poly-viral coinfections and disease severity, or if coinfection rates were different between pre and post-pandemic periods [35]. Despite possible limitations, our findings show robust data over a period of 5 years and add to the current body of literature on RSV epidemics.

## Conclusion

In conclusion, we showed the epidemiologic trends of RSV infections and hospitalizations over a 5-year period in a hospital in Italy, and we developed a forecasting model to predict the peak of hospitalizations for the next RSV epidemic season. We show the potential of combining forecasting models as the one we developed with detailed surveillance data. Implementing such systems would help allocating hospital resources and ensure proper preparedness for future RSV epidemics [45].

### Electronic supplementary material

Below is the link to the electronic supplementary material.

Supplementary Material 1. ICD 9-CM diagnosis at ED discharge for ARI

Supplementary Material 2. Trend of laboratory-confirmed RSV infections by season and age-classes; OPBG, January 2018– December 2022

Supplementary Material 3. Trend of RSV-related hospitalizations by season and age-classes; OPBG, January 2018– December 2022

Supplementary Material 4. Trend of ICU admissions by season and age-classes; OPBG, January 2018– December 2022

## Data Availability

The original contributions presented in the study are included in the article/supplementary file, further inquiries can be directed to the corresponding author/s.

## Abbreviations

RSV: Respiratory Syncytial Virus
SARIMA: Seasonal AutoRegressive Integrated Moving Average
PCR: Polymerase Chain Reaction
CDC: Centers for Disease Control and Prevention
NREVSS: National Respiratory and Enteric Virus Surveillance System
ED: Emergency department
HEIS: Healthcare Emergency Information System
ARI: Acute Respiratory Infections
ICU: Intensive Care Unit
AIC: Akaike Information Criterion
BIC: Bayesian Information Criterion
MAPE: Mean Absolute Percentage Error
OPBG: Ospedale Pediatrico Bambin Gesu
