# Supplementary material for: Epidemiology of respiratory syncytial virus in a large pediatric hospital in Central Italy and development of a forecasting model to predict the seasonal peak
Source: Ital J Pediatr. 2024 Apr 8;50:65. doi: 10.1186/s13052-024-01624-x (PMC11003041; doi:10.1186/s13052-024-01624-x)
Supplement: Supplementary file 1 — Supplementary Material 1. ICD 9-CM diagnosis at ED discharge for ARI [file 13052_2024_1624_MOESM1_ESM.pdf]

Supplementary file 1. ICD 9-CM diagnosis at ED discharge for ARI

**ICD-9 CM code and description**
460 ACUTE NASOPHARYNGITIS
461 AC SINUSITIS
4610 AC MAXILLARY SINUSITIS
4611 AC FRONTAL SINUSITIS
4612 AC ETHMOIDAL SINUSITIS
4613 AC SPHENOIDAL SINUSITIS
4618 OTHER ACUTE SINUSITIS
4619 ACUTE SINUSITIS NOS
462 ACUTE PHARYNGITIS
463 ACUTE TONSILLITIS
4640 AC LARYNGITIS
46400 AC LARYNGITIS W/O OBST
46401 AC LARYNGITIS W OBSTRUCT
46410 AC TRACHEITIS NO OBSTRUC
46411 AC TRACHEITIS W OBSTRUCT
4642 AC LARYNGOTRACH
46420 AC LARYNGOTRACH NO OBSTR
46421 AC LARYNGOTRACH W OBSTR
4643 AC EPIGLOTTITIS
46430 AC EPIGLOTTITIS NO OBSTR
46431 AC EPIGLOTTITIS W OBSTR
4644 CROUP
4659 ACUTE URI NOS
4660 ACUTE BRONCHITIS
46611 ACU BRONCHOLITIS D/T RSV
46619 ACU BRNCHLTS D/T OTH ORG
4800 ADENOVIRAL PNEUMONIA
4801 RESP SYNCYT VIRAL PNEUM
4802 PARINFLUENZA VIRAL PNEUM
4808 VIRAL PNEUMONIA NEC
4809 VIRAL PNEUMONIA NOS
481 PNEUMOCOCCAL PNEUMONIA
48283 PNEUMO OTH GRM-NEG BACT
48289 PNEUMONIA OTH SPCF BACT
4829 BACTERIAL PNEUMONIA NOS
4830 PNEU MYCPLSM PNEUMONIAE
4838 PNEUMON OTH SPEC ORGNSM
485 BRONCHOPNEUMONIA ORG NOS
486 PNEUMONIA, ORGANISM NOS
4870 INFLUENZA WITH PNEUMONIA
4871 FLU W RESP MANIFEST NEC
4878 FLU W MANIFESTATION NEC
7806 FEVER

78606 TACHYPNEA
78609 RESPIRATORY ABNORM NEC
7862 COUGH
78650 CHEST PAIN NOS
78651 PRECORDIAL PAIN
78652 PAINFUL RESPIRATION
78659 CHEST PAIN NEC
7931 ABN FINDINGS-LUNG FIELD
