# Supplementary material for: Epidemiology of respiratory syncytial virus in a large pediatric hospital in Central Italy and development of a forecasting model to predict the seasonal peak
Source: Ital J Pediatr. 2024 Apr 8;50:65. doi: 10.1186/s13052-024-01624-x (PMC11003041; doi:10.1186/s13052-024-01624-x)
Supplement: Supplementary file 2 — Supplementary Material 2. Trend of laboratory-confirmed RSV infections by season and age-classes; OPBG, January 2018– December 2022 [file 13052_2024_1624_MOESM2_ESM.pdf]

**Supplementary file 2. Trend of laboratory-confirmed RSV infections by season and age-classes; OPBG, January 2018 – December 2022**

| | **Season 2017-2018** | **Season 2018-2019** | **Season 2019-2020** | **Season 2020-2021** | **Season 2021-2022** | **Season 2022-2023** | **Total** | ***P-value*** |
|---|---|---|---|---|---|---|---|---|
| **Age classes** | | | | | | | | |
| **<1** | 322 (67.9) | 524 (66.6) | 451 (65.6) | 2 (40.0) | 529 (55.3) | 288 (61.8) | **2,116 (62.6)** | <0.001 |
| **1-4** | 99 (20.9) | 196 (24.9) | 188 (27.3) | 2 (40.0) | 323 (33.7) | 126 (26.8) | **934 (27.6)** | <0.001 |
| **5-9** | 30 (6.3) | 40 (5.1) | 25 (3.6) | 1 (20.0) | 65 (6.8) | 34 (7.3) | **195 (5.8)** | 0.04 |
| **≥10** | 23 (4.8) | 27 (3.4) | 24 (3.5) | 0 (0.0) | 40 (4.2) | 19 (4.1) | **133 (3.9)** | 0.7 |
| **Total** | **474 (100)** | **787 (100)** | **688 (100)** | **5 (100)** | **957 (100)** | **466 (100)** | **3,378 (100)** | |

*Seasons 2017-2018 consider the first 12 weeks of the year 2018 and the last 12 of the year 2022; the other seasons were completed and lasted from from week 39 of the previous year and ending at week 12 of the next year*
