# Supplementary material for: Epidemiology of respiratory syncytial virus in a large pediatric hospital in Central Italy and development of a forecasting model to predict the seasonal peak
Source: Ital J Pediatr. 2024 Apr 8;50:65. doi: 10.1186/s13052-024-01624-x (PMC11003041; doi:10.1186/s13052-024-01624-x)
Supplement: Supplementary file 3 — Supplementary Material 3. Trend of RSV-related hospitalizations by season and age-classes; OPBG, January 2018– December 2022 [file 13052_2024_1624_MOESM3_ESM.pdf]

Supplementary file 3. Trend of RSV-related hospitalizations by season and age-classes; OPBG, January 2018 – December 2022

	Season 2017-2018	Season 2018-2019	Season 2019-2020	Season 2020-2021	Season 2021-2022	Season 2022-2023	Total	*P-value*
**Age classes**								
**<1**	234 (88.6)	394 (85.3)	361 (86.6)	2 (100.0)	286 (81.7)	251 (72.7)	**1,528 (83.0)**	<0.001
**1-4**	24 (9.1)	56 (12.1)	52 (12.5)	0 (0.0)	51 (14.6)	76 (22.0)	**259 (14.1)**	<0.001
**5-9**	5 (1.9)	10 (2.2)	3 (0.7)	0 (0.0)	9 (2.6)	11 (3.2)	**38 (2.1)**	0.3
**≥10**	1 (0.4)	2 (0.4)	1 (0.2)	0 (0.0)	4 (1.1)	7 (2.0)	**15 (0.8)**	0.02
**Total**	**264 (100)**	**462 (100)**	**417 (100)**	**2 (100)**	**350 (100)**	**345 (100)**	**1,840 (100)**	

Seasons 2017-2018 consider the first 12 weeks of the year 2018 and the last 12 of the year 2022; the other seasons were completed and lasted from from week 39 of the previous year and ending at week 12 of the next year
