# Supplementary material for: Epidemiology of respiratory syncytial virus in a large pediatric hospital in Central Italy and development of a forecasting model to predict the seasonal peak
Source: Ital J Pediatr. 2024 Apr 8;50:65. doi: 10.1186/s13052-024-01624-x (PMC11003041; doi:10.1186/s13052-024-01624-x)
Supplement: Supplementary file 4 — Supplementary Material 4. Trend of ICU admissions by season and age-classes; OPBG, January 2018– December 2022 [file 13052_2024_1624_MOESM4_ESM.pdf]

Supplementary file 4. Trend of ICU admissions by season and age-classes; OPBG, January 2018 – December 2022

	Season 2017-2018	Season 2018-2019	Season 2019-2020	Season 2020-2021	Season 2021-2022	Season 2022-2023	Total	*P-value*
**Age classes**								
**<1**	34 (94.4)	52 (96.3)	38 (95.0)	1 (100)	38 (90.5)	46 (84.1)	**209 (91.3)**	<0.001
**1-4**	2 (5.6)	2 (3.7)	2 (5.0)	0 (0.0)	3 (7.1)	9 (16.1)	**18 (7.9)**	0.02
**5-9**	0 (0.0)	0 (0.0)	0 (0.0)	0 (0.0)	1 (2.3)	1 (1.8)	**2 (0.9)**	-
**≥10**	0 (0.0)	0 (0.0)	0 (0.0)	0 (0.0)	0 (0.0)	0 (0.0)	**0 (0.0)**	-
**Total**	**36 (100)**	**54 (100)**	**40 (100)**	**1 (100)**	**42 (100)**	**56 (100)**	**229 (100)**	

Seasons 2017-2018 consider the first 12 weeks of the year 2018 and the last 12 of the year 2022; the other seasons were completed and lasted from from week 39 of the previous year and ending at week 12 of the next year
